# A Twist in the Tale: An Unexpected Pathogen in a Patient With a Cavitary Lung Lesion

**DOI:** 10.7759/cureus.85511

**Published:** 2025-06-07

**Authors:** Thomas Van Den Bosch, Benedicte De Muynck, Peter Bomans, Nikki Granacher

**Affiliations:** 1 Pulmonology, Ziekenhuis aan de Stroom, Antwerp, BEL; 2 Hematology, Ziekenhuis aan de Stroom, Antwerp, BEL

**Keywords:** cavitating pneumonia, false-positive galactomannan, immunocompromised patient, legionella pneumophila, lung abscess

## Abstract

We present a challenging case of *Legionella pneumophila *lung abscess in a 58-year-old woman with myelodysplastic syndrome presenting with a cavitary lung lesion. The diagnostic workup was complicated by a false-positive galactomannan result, resulting in unnecessary medicamentous treatment and possible different therapeutic decisions. Management involved surgical resection regarding a necessary stem cell transplant and prolonged antibiotic (with monotherapy versus broad-spectrum) and antifungal therapy. This case highlights the need to consider atypical pathogens in immunocompromised patients, even when other findings suggest more common infections, and underscores the importance of a multidisciplinary approach and careful interpretation of laboratory results to guide appropriate management strategies.

## Introduction

*Legionella pneumophila *is a known cause of atypical pneumonia, often presenting with systemic symptoms and bilateral infiltrates. However, it is a rare cause of lung abscess, making such presentation an uncommon clinical manifestation [1]. Diagnosis can be challenging due to the fastidious nature of the organism [2,3] and the potential for false-positive results with other diagnostic tests, such as the galactomannan assay [4]. Due to the patient's immunocompromised status, it is essential to consider both typical, atypical, and opportunistic causes of a lung abscess [5].

We present a case of *Legionella* lung abscess in an immunocompromised patient with myelodysplastic syndrome, complicated by a false-positive *Aspergillus galactomannan* antigen test, to illustrate the diagnostic difficulties and management considerations in such cases.

## Case presentation

A 58-year-old woman with a history of myelodysplastic syndrome of less than one year's duration before presentation was scheduled for allogeneic stem cell transplantation due to progressive transfusion dependence. She was immunocompromised with dysplastic white blood cells and blasts found in the bone marrow analysis. She had recently been diagnosed with a deep vein thrombosis in the left gastrocnemius vein and started on direct oral anticoagulant (DOAC) therapy.

**Figure 1:**
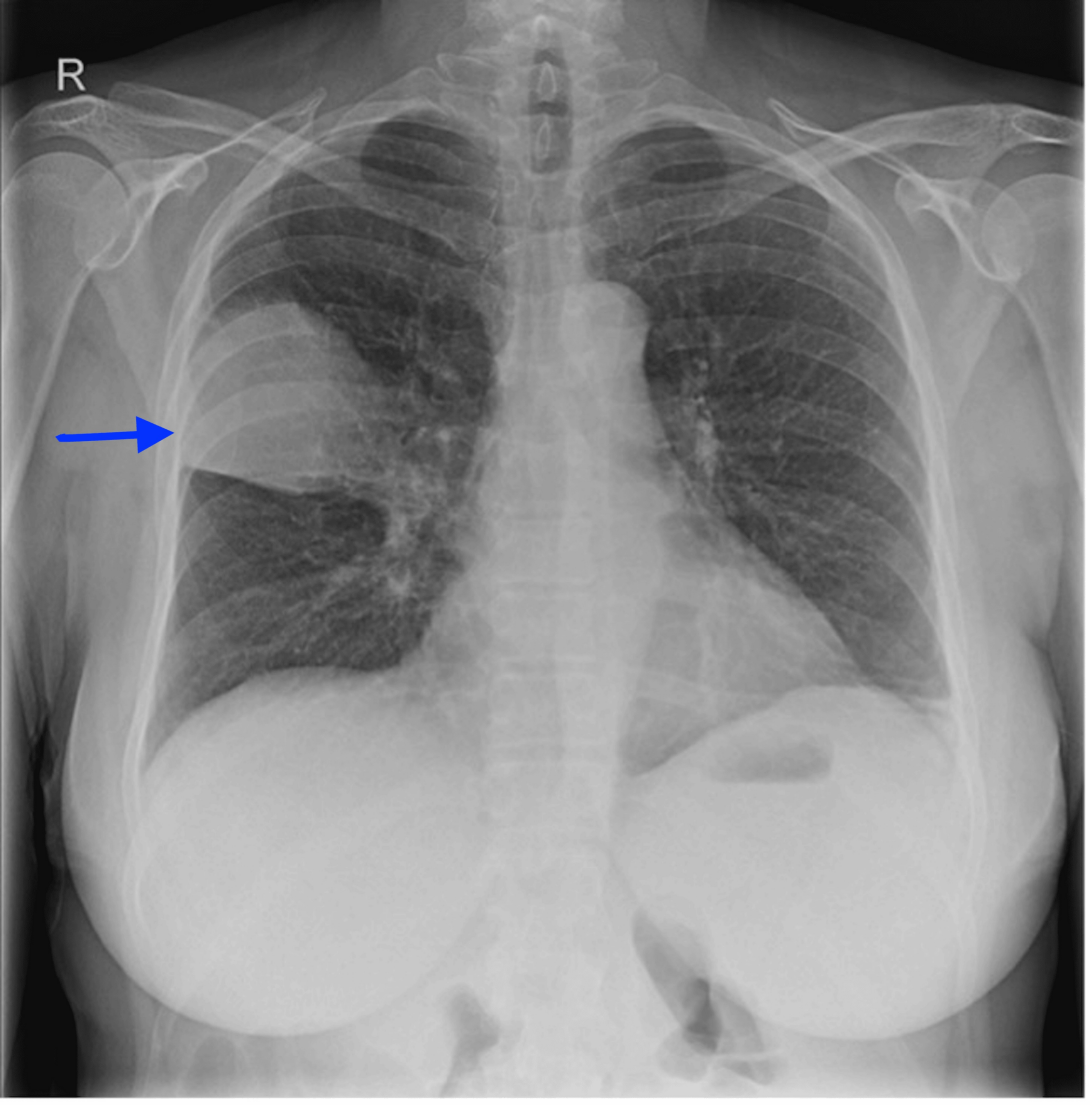
X-ray at presentation shows a consolidation in the right upper lobe

Four weeks prior to referral to the pulmonology department, she was admitted to the hematology department for treatment of right upper lobe pneumonia (Figure 1). She received antibiotic treatment with piperacillin-tazobactam for seven days. No pathogen was identified; sputum culture, respiratory panel on nasopharyngeal aspirate, including polymerase chain reaction (PCR) for *Legionella pneumophila *and *Mycoplasma pneumoniae*, and urinary *Legionella *antigen (only possible detection of serogroup 1) were all negative. Her C-reactive protein (CRP) normalized from 375 mg/L to 9 mg/L after this treatment. Because of the good biochemical response, no follow-up imaging was performed.

Three weeks after discharge, she presented again to the hematology clinic with persistent daily fevers in the evening and night sweats. She reported no other symptoms and was admitted to the pulmonology service.

On initial evaluation by the pulmonology service, the patient was febrile (38.2°C) with an asymptomatic low blood pressure of 107/62 mmHg and a regular heart rate. She was not in respiratory distress. Lung auscultation revealed decreased breath sounds on the right side. No cervical lymphadenopathy was noted. As additional information, the patient stayed at a wellness hotel (where she visited multiple times a sauna and swimming pool) 10 days before the start of the pneumonia.

**Figure 2:**
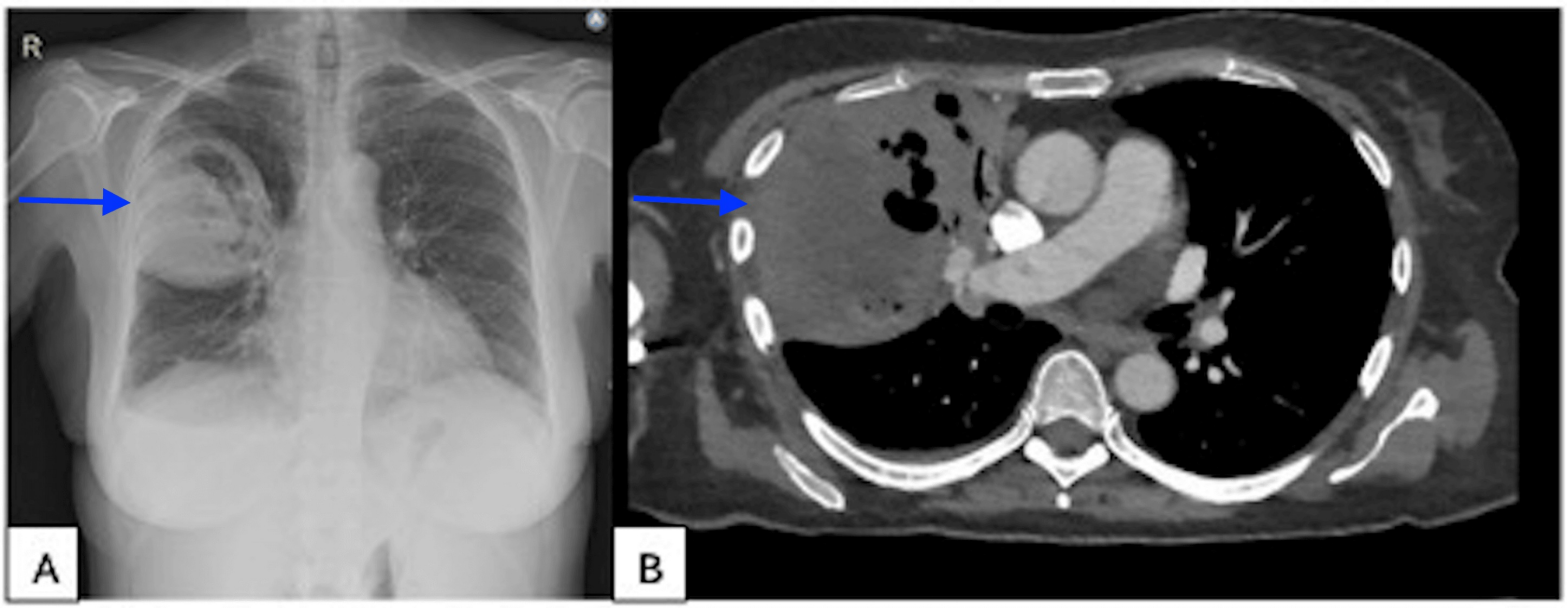
(A) X-ray after initial treatment shows cavitation of the initial consolidation four weeks after start treatment. (B) CT scan of the thorax confirms these finding more in detail: a thick-walled cavity with a diameter of 9 cm and air-fluid levels CT: computed tomography

Laboratory results showed a mildly elevated CRP of 23 mg/L (reference range: <5 mg/L) without leukocytosis. Monocytosis, anemia, and thrombocytopenia were noted, consistent with the myelodysplastic syndrome. A chest radiograph four weeks after the initial chest radiograph showed a cavitary lesion in the right lung. Chest CT on the same day confirmed the presence of a cavity in the right upper lobe (Figure 2).

Bronchoscopy following the CT findings revealed no endobronchial abnormalities. Bronchoalveolar lavage (BAL) fluid analysis, including extensive microbiological testing, was positive for *Legionella pneumophila *by PCR. All cultures were negative, and further microbiological analysis, including tests for mycobacterial and fungal infections with galactomannan on BAL fluid, yielded no additional positive findings.

Outpatient treatment with levofloxacin was initiated following three days of empiric treatment with amoxicillin-clavulanic acid, which had been started immediately after the bronchoscopy. After one week, her symptoms persisted, and she was readmitted with the start of intravenous antibiotic therapy with piperacillin-tazobactam. Levofloxacin was converted to moxifloxacin in consultation with infectious disease, based on the local susceptibility of *Legionella* species. A repeat chest CT after seven days of treatment with piperacillin-tazobactam and moxifloxacin showed enlargement of the lung abscess. Nevertheless, CRP remained low (15.2 mg/L).

A second bronchoscopy was performed, which was complicated by bleeding from the right upper lobe despite interruption of her DOAC for 48 hours prior to bronchoscopy and despite transfusion of blood platelets before bronchoscopy. Because of the easy bleeding despite correction of platelets and adequate pausing of her DOAC, the suspicion of a possible angio-invasive aspergillosis rose. Antibiotic therapy was empirically escalated to treatment with meropenem and with continuation of moxifloxacin. The bronchoscopy confirmed the positive PCR for *Legionella pneumophila*, but also an elevated galactomannan value (2.7) was detected in BAL fluid. Voriconazole was initiated in the patient, who was considered high risk for invasive fungal disease based on clinical findings, imaging, and microbiological criteria, despite not fully meeting the European Organization for Research and Treatment of Cancer/Mycoses Study Group criteria (EORTC/MSG) for probable invasive aspergillosis. BAL culture remained negative for *Aspergillus* species. *Aspergillus* antigen in blood was not elevated. The patient was transferred to the intensive care unit for an upgrade of care because of a higher oxygen need (high-flow nasal cannula) after the endobronchial bleeding.

Following extensive multidisciplinary discussion, a surgical approach with bilobectomy (right upper and middle lobes) was pursued. A significant dilemma existed regarding the optimal management strategy. While stem cell transplantation was planned, prolonged medical therapy for the lung abscess alone would have unacceptably delayed this life-saving intervention, potentially leading to a fatal outcome. She carried a risk of acute leukemia of 21% in the following four years, with a median survival of 37 months, given an intermediate-2 chronic myelomonocytic leukemia-specific prognostic scoring system-Mol risk group.

Conversely, surgical resection of the abscess offered the possibility of accelerating the healing process of the lung abscess and facilitating timely stem cell transplantation. However, this approach carried a higher mortality risk given the patient's underlying myelodysplastic syndrome and associated comorbidities. Despite negative fungal cultures, the causative agent of the lung abscess was still considered to be *Aspergillus*, further complicating the decision-making process. Ultimately, the decision was made to proceed with bilobectomy to expedite the patient's recovery and enable the planned stem cell transplantation and prevent the risk of progression of the myelodysplastic syndrome to acute leukemia.

Pathology of the resected lobes was surprising: no hyphae were observed, and no fungi were cultured. Grocott methenamine silver stain remained negative. However, PCR for *Legionella *was again positive on lung biopsy. Histological specimens were sent for culture, yielding *Legionella pneumophila *serotype 2-15. All other cultures, including fungal and mycobacterial analysis, remained negative.

The bilobectomy was complicated by pulmonary vein bleeding, leading to acute respiratory distress syndrome and multiple organ failure (acute kidney injury requiring dialysis and hemodynamic failure resulting in vasopressor dependence). A repeat bronchoscopy with BAL was performed to optimize pulmonary condition and to culture bronchial aspirates, showing no evidence of *Aspergillus* infection (negative fungal cultures and, surprisingly, a negative galactomannan on BAL this time).

Weaning from mechanical ventilation was not possible, and two weeks post-bilobectomy, the patient developed ventilator-associated pneumonia. She rapidly deteriorated with refractory multiple organ failure and, unfortunately, passed away.

**Table 1:** Timeline and important (positive) findings CRP: C-reactive protein, CT: computed tomography, BAL: bronchoalveolar lavage, PCR: polymerase chain reaction

Day	-10	0	7	21	27	35	54
Event	Holiday wellness center	Admission ward hematology	Discharge ward hematology	Outpatient visit to hematology/pulmonology	Admission ward hematology	Bilobectomy	Death
CRP (mg/L)	-	375	9	23	15.2	34	-
X-ray	-	Lobar pneumonia (Figure 1)	-	Cavitary lesion at the same location as the lobar pneumonia (Figure 2)	Enlargement of the cavitary lesion on chest CT	-	-
Positive findings	-	None	-	PCR *Legionella* on BAL fluid	PCR *Legionella* on BAL, *Aspergillus* antigen test positive on BAL (2.7)	Culture *Legionella* positive	-
Treatment	-	Piperacilline-tazobactam (7 days)	-	Amoxicillin-clavulanic acid for 3 days, followed by levofloxacin monotherapy for 5 days	Switch to piperacilline, tazobactam, moxifloxacin, and voriconazole	Continuation antimicrobial therapy	-

## Discussion

*Legionella*, while a rare cause of lung abscess, should be considered in the differential diagnosis of cavitary lung lesions, especially in immunocompromised patients, such as those with hematologic malignancies or those receiving high-dose corticosteroids [1,2,5]. The atypical radiographic presentation of *Legionella* (cavitary lung lesion) is associated with the more difficult-to-diagnose non-pneumophila species and has an indolent or asymptomatic clinical presentation [1,5]. The diagnosis can be challenging due to the fastidious nature of the organism, which requires specialized culture techniques. Additionally, the fact that a pulmonary abscess is mostly caused by a polymicrobial infection makes this case unusual. PCR testing has improved the sensitivity and speed of diagnosis. As *Legionella* serotype 1 is the most prevalent causative agent for infections and detected with a urinary antigen, PCR testing can detect different *Legionella* serogroups (or *Legionella* species) [1-3,6].

Given the temporal relationship of visiting a hotel with wellness prior to the development of pneumonia, we consider *Legionella* as the causative agent of the disease. Probably, there was a bacterial coinfection, given the good response of biochemical inflammatory parameters. The fact that *Legionella* was not initially identified is due to testing with only the urinary antigen. The ongoing infection likely contributed to the progressive disease, despite broad-spectrum antibiotic therapy, due to *Legionella pneumophila* serogroup 2-15, which was not covered by the antibiotic regimen.

Treatment of *Legionella* lung abscess typically involves prolonged antibiotic therapy. Macrolides and fluoroquinolones are commonly used, although the optimal duration and combination of antibiotics are subject to ongoing debate. Some evidence suggests that monotherapy may be as effective as combination therapy, with the potential advantage of reduced toxicity and drug interactions. Anaerobic coverage should be considered, especially in cases of lung abscess [2]. In our case, monotherapy was chosen after the diagnosis of *Legionella* infection because the patient had already received broad-spectrum antibiotic therapy prior to this.

Invasive aspergillosis is a serious concern in immunocompromised individuals, particularly those undergoing stem cell transplantation. Diagnosis relies on a combination of clinical, radiological, and laboratory findings, including galactomannan and PCR testing [7]. Antifungal therapy is indicated for confirmed or probable invasive aspergillosis [7]. The EORTC/MSG criteria provide a standardized approach to classifying the probability of invasive fungal disease [8,9]. However, this case highlights the potential for discrepancies between clinical and microbiological findings and the importance of integrating all available data when making a diagnosis. False-positive galactomannan results can lead to misdiagnosis and unnecessary antifungal therapy, underscoring the importance of carefully interpreting diagnostic tests in immunocompromised patients [4]. When such discrepancies arise, multidisciplinary discussion can be helpful.

False-positive galactomannan results can occur due to several reasons, such as fungal infections other than *Aspergillus* infections, bacterial infections (e.g., nocardiosis) [10], medicamentous (certain antifungals and antibiotics, e.g., certain brands of piperacillin-tazobactam) [11,12], and colonization with *Aspergillus* without infection (especially in immunocompromised patients) [4]. These factors highlight the importance of considering alternative diagnoses, such as *Legionella*, when interpreting a positive galactomannan result, especially in patients with risk factors for *Legionella* infection. The positive predictive value of galactomannan is known to be higher in patients with hematologic malignancies, further complicating interpretation in this patient population [4].

## Conclusions

In this report, we describe a complex case of *Legionella *lung abscess and discuss the diagnostic challenges encountered, particularly the potential for false-positive galactomannan results to confound the evaluation. This experience underscores several important considerations in the management of cavitary lung lesions. First, while rare, *Legionella *should be considered, particularly in immunocompromised patients with predisposing factors. Second, a thorough history focusing on potential *Legionella *exposure is crucial. Third, appropriate diagnostic testing, including deep respiratory sampling and the use of specialized culture techniques in combination with PCR for *Legionella*, is essential for accurate diagnosis. Fourth, while optimal antibiotic therapy remains a topic of debate, anaerobic coverage should be considered in cases of pulmonary abscesses. Finally, clinicians must be aware of the potential for false-positive galactomannan results and avoid unnecessary antifungal treatment. Repeat testing, using different diagnostic modalities (e.g., culture, antigen testing, PCR), and applying the EORTC/MSG criteria to the correct population (patients with cancer and stem cell or solid-organ transplant patients) can be helpful.
